# Popliteal fossa reconstruction with medial genicular artery flap in a low resource setting: A report of two cases

**DOI:** 10.1016/j.ijscr.2020.02.056

**Published:** 2020-02-28

**Authors:** U.U. Nnadozie, C.C. Maduba

**Affiliations:** a Department of Surgery, Ebonyi State University, Abakaliki, Ebonyi State, Nigeria; b Division of Plastic Surgery, Department of Surgery, Alex Ekwueme Federal University Teaching Hospital, Abakaliki, Ebonyi State, Nigeria

**Keywords:** Medial-genicular-artery-flap, Popliteal-fossa, Reconstruction, Case-report

## Abstract

**Background:**

Popliteal fossa defects are common arising from several causes. Options of reconstruction around the knee could be limited by the cause of defect or interventions. Medial genicular artery flap is known in the books but not in popular use despite its obvious advantages of superior vascularity, adequate size, suppleness, and hidden donor site.

**Aim:**

To promote the use of this flap due to its advantages and ease of use especially in resource poor settings.

**Patients and methods:**

We report two patients from a low resource setting aged 23 and 20 years respectively. The first case was managed for avulsion wound of the popliteal fossa while the second had post burn knee contracture release. The resultant large popliteal fossa defects on both patients were seen on clinical examination. Both patients were offered popliteal fossa reconstruction for the popliteal fossa defects using medial genicular artery flap with good outcome.

**Conclusion:**

The medial genicular artery flap is a veritable option of popliteal fossa reconstruction especially for defects that are located contiguous to the flap and when other regional flap options are not available. Flap survival is excellent and donor site is hidden.

## Highlights

• Large popliteal fossa defects require coverage with pliable tissues.
• Medial genicular artery flap satisfies this requirement.
• In a low income setting like ours, this flap is a veritable option.

## 1 Introduction

Popliteal fossa is a diamond shaped anatomical shallow depression located at the back of the knee joint. Defects of the popliteal fossa includes the traumatic, post-burn contracture release, infective and oncologic causes [1]. These defects are usually very challenging to reconstruct due to exposure of the osteo-articular, tendino-muscular and the neurovascular structures around the joint [1]. When the defects are allowed to heal secondarily the resulting scars have a lot of consequences such as infection, desiccation of joint structures, implant loss and re-contracture [2,3]. Several options have been devised therefore to reconstruct these defects ranging from the simple local flaps, through regional muscle and fascio-cutaneous flaps, to complex micro-surgical free flaps [4].

The goal of reconstruction is to restore both knee contour and preserve knee function. This could be achieved with variable options of which the use of medial genicular artery flap is one. This flap could be a preferred choice when other regional flaps are not available and when the defect location is more suitably handled with it [5]. The use of medial genicular flaps when available and suitable is encouraged because it is simple, single-staged, technically easy and sizeable with an easily hidden secondary defect. It is also thin and therefore maintains the contour of the joint [2,5,6].

This flap is not widely reported in published work for popliteal fossa reconstruction and sketchily mentioned alongside the lateral genicular artery counterpart in the encyclopedia of flaps [5]. However it has since been shown from cadaveric dissection that there is a consistent pattern of blood supply to the flap and safety of flap elevation beyond the midpoint of the thigh [6]. There are reports of its use in infrapatellar and patellar knee defects [7]. We are not aware of any reports indicating its particular use for popliteal fossa reconstruction.

In our study we report our experience with two patients in which large popliteal soft tissue defects were successfully managed with medial genicular artery flap with good outcome. The case report of these two patients was written in line with SCARE criteria [8].

Our hospital, Alex Ekwueme Federal University Teaching Hospital Abakaliki (AEFUTHA) is a 720 bed capacity tertiary health care facility located in the center of Abakaliki, the capital of Ebonyi State in Nigeria, a low income setting.

## 2 Patients and method

**Fig. 1:**
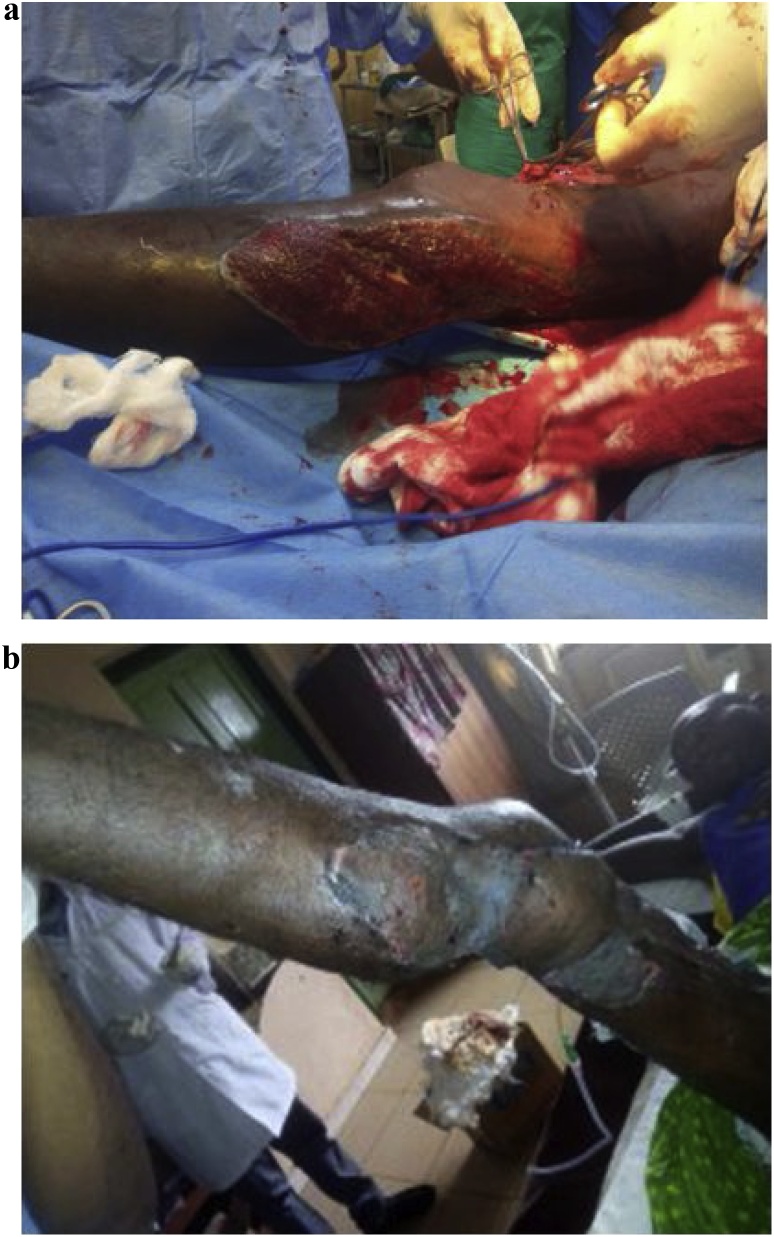
a) Left popliteal fossa avulsion (pre-operative). b) Left popliteal fossa avulsion (post-operative).

Two patients used for this study were managed in the unit about six months apart. The first patient had an avulsion wound over the left popliteal fossa exposing the muscle following road traffic accident (Fig. 1a). He was a 23 year old male student admitted through accident and emergency unit of Alex Ekwueme Federal University Teaching Hospital Abakaliki (AEFUTHA) with significant blood loss. The avulsion wound involved the popliteal fossa extending to the posterior border of the proposed flap making it a favorable option. His haemogram and serum electrolytes were within normal limits. Patient had debridement and was commenced on dressing with honey and 5% povidone iodine at different times. He was later worked up for popliteal fossa resurfacing with medial genicular artery flap about 8 days after injury. Flap was raised under spinal anesthesia and tourniquet. Flap was planned in reverse and about 16 × 10 cm flap raised as fasciocutaneous tissue designed about the midpoint of an imaginary line joining the midpoint of inguinal ligament to the medial femoral condyle as the axis. Flap was inset on the defect with vicryl sutures over a Redivac drain. Secondary defect was resurfaced with split-thickness skin grafting. Nearly 100% flap survival was achieved except for a slight marginal tip necrosis that healed with dressing (Fig. 1b). Knee function was satisfactory and patient was discharged to continue follow-up on out-patient basis.

**Fig. 2:**
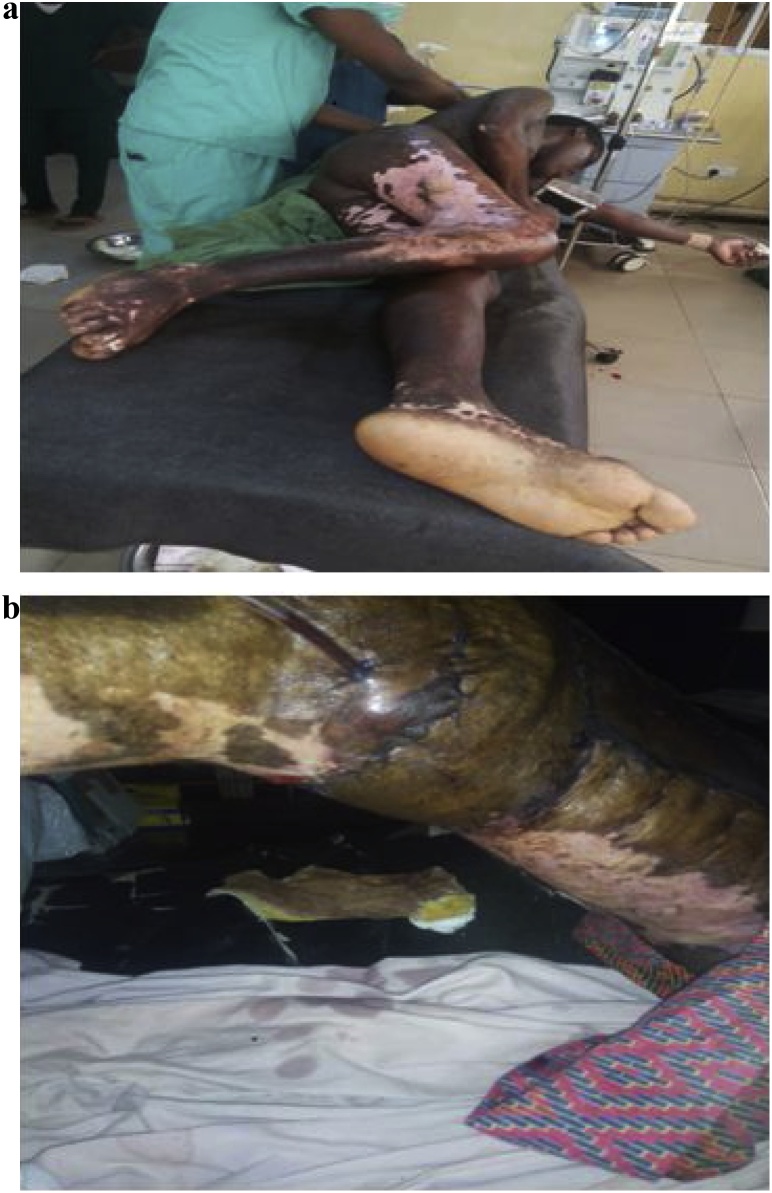
a) Post burn knee contracture (pre-operative). b) Post burn knee contracture release (post-operative).

The second patient was a 20 year old out of school male with right knee contracture in 90 degrees fixed flexion of about 1 year following a poorly managed major flame burns (Fig. 2a). He was managed by medical officers in a general hospital without any form of splinting or physiotherapy during seven month admission. He had no chronic medical condition and does not have any history of drug allergy. His haemogram and electrolytes were within normal limits.

He had good clinical and laboratory parameters. The right knee was on 90° flexion deformity. The lateral surface of the thigh had extensive ulcerated unstable scar covering the territory of the lateral genicular artery flap. There were hyperpigmented mature scars on the trunk and upper extremities. He was admitted to the ward and worked up for contracture release and popliteal fossa reconstruction with medial genicular artery flap. Surgery was done by the consultant plastic surgeon under subarachnoid block. The knee was released up to 165°.

The flap was designed along an imaginary axis joining the medial femoral condyle and the midpoint of inguinal ligament. The flap size was 16 × 8 cm planned in reverse. It was raised as a fasciocutaneous flap and inset on the popliteal defect with two layer suturing. Conventional gauze dressing was used and plaster of Paris back-slab applied to maintain immobilization at 165°. Flap survival was very good without any necrosis (Fig. 2b). Patient had alternate day wound dressing with povidone iodine soaked gauze of the flap and the split-thickness skin graft recipient site his till discharge. Physiotherapy was commenced after two weeks and eventual knee extension was 175°. Patient was satisfied and walked home initially with the aid of a walking stick and subsequently without any support.

## 3 Discussion

Large popliteal fossa defects like other knee defects require coverage with flaps which when thin and pliable give, in addition to functional cover, a good aesthetic outcome [9]. Medial genicular artery flap satisfies all these requirements and does not leave morbidity of function that is associated with muscle flap, being a fasciocutaneous flap [9,10]. It is useful in settings in which other regional flaps are not available or in which the defect is more contiguous to the flap [5]. It is a versatile flap for coverage of defects around the knee. We found it particularly applicable in management of popliteal fossa defect based on the above two criteria. In the first patient the defect is contiguous to the postero-medial border of the flap; while in the second case it was more feasible to use than the other regional flaps which were affected by burns scarring.

Pedicle flaps remain a workhorse in resource poor countries and readily resorted to for the technical ease and affordability [2]. Use of medial genicular artery flap therefore is favored in such setting. Both trauma and post burn contractures of the knee constitute a significant proportion of the defect around the knee especially in the developing countries [2,9]. Reconstruction with this flap will contribute significantly in managing the burden of wounds of the popliteal fossa when properly selected.

More so, flap survival is very good owing to rich vascular network that sustains the flap [6]. This was consistent with our experience in which the flap survival was essentially 100% except for insignificant marginal necrosis that healed with dressing in the first case. Post-operative management was also not technically demanding as patients were nursed in general plastic ward.

The functional recovery was satisfactory. However we observed that the first patient who was of a higher socio-economic background had had more adipose tissue in the flap. The flap was therefore bulkier than that of the second patient. It gave better padding but was cosmetically inferior to the flap in the second patient which was thinner and better fitted to contour of the popliteal fossa. This bulkiness was also observed in female patients in a reported work where the bulkiness was due to greater adipose tissue thickness in females [2].

Our patients were satisfied with outcome and duration of hospital stay which were respectively 21 days and 30 days in the first and second cases. The good flap healing, the ease of nursing and ultimately the hidden donor site all contributed to the feeling of satisfaction.

The first patient had no range of motion challenges pre-operatively as well as post-operatively. The second patient however had limitation of extension which eventually improved from 165° to 175° following physiotherapy. However the second patient continues on physiotherapy on out-patient basis.

## 4 Conclusion

Medial genicular artery flap is a reliable easy to raise option for resurfacing of popliteal fossa defects with supple, stable padding that is aesthetically satisfactory and with hidden donor site. It is resorted to in settings where the defect is contiguous to the flap and where other flap options may not be readily available. In the resource poor nations where the pedicle flaps are the still the mainstay of treatment, it is a reliable workhorse for popliteal fossa coverage. The two cases reviewed in this article were respectively for the above two reasons.

## Sources of funding

We did not get any form of funding from any person, organisation or institutions.

## Ethical approval

We obtained ethical approval from the Ethical Committee of Alex Ekwueme Federal University Teaching Hospital Abakaliki for the study.

The approval reference number is REC APPROVAL NUMBER 06/11/2019-08/11/2019.

## Consents

Written informed consent was obtained from the patients for publication of this case reports and accompanying images.

## Author contribution

Dr. Ugochukwu Uzodimma Nnadozie: Conceptualization, Literature review, Methodology, software, Manuscript writing, Editing and Proof reading, final approval of the manuscript and Submission.

Dr. Charles Chidiebele Maduba: Conceptualization, Literature review, Methodology, Software, Manuscript writing, Editing and Proof reading and final approval of the manuscript.

## Registration of research studies

Not applicable to this study.

## Guarantor

Dr Ugochukwu Uzodimma Nnadozie.

## Provenance and peer review

Editorially reviewed, not externally peer-reviewed.

## Declaration of Competing Interest

We declare that we have no conflict of interest in this study.
